# Selective cancer cell killing by α-tocopheryl succinate

**DOI:** 10.1054/bjoc.2000.1559

**Published:** 2001-01-01

**Authors:** J Neuzil, T Weber, N Gellert, C Weber

**Affiliations:** Institute for Prevention of Cardiovascular Diseases, Ludwig Maximilians University, Pettenkoferstrasse 9, Munich, 80336, Germany

**Keywords:** vitamin E analogue, malignant cells, apoptosis, selectivity

## Abstract

We report that α-tocopheryl succinate, a vitamin E analogue with pro-apoptotic properties, selectively kills cells with a malignant or transformed phenotype, i.e. multiple haematopoietic and carcinoma cell lines, while being non-toxic to normal, i.e. primary and non-transformed cells. These findings strongly suggest a potential of this micronutrient in the therapy and/or prevention of cancer without significant side-effects. © 2001 Cancer Research Campaign http://www.bjcancer.com


*British Journal of Cancer* (2000) **84**(1), 87–89
© 2001 Cancer Research Campaign
doi: 10.1054/ bjoc.2000.1559, available online at http://www.idealibrary.com on IDEAL®

http://www.bjcancer.com

Short Communication

### Selective cancer cell killing by α-tocopheryl succinate

**J Neuzil, T Weber, N Gellert and C Weber**

Institute for Prevention of Cardiovascular Diseases, Ludwig Maximilians University, Pettenkoferstrasse 9, 80336 Munich, Germany

**Summary** We report that α-tocopheryl succinate, a vitamin E analogue with pro-apoptotic properties, selectively kills cells with a malignant or transformed phenotype, i.e. multiple haematopoietic and carcinoma cell lines, while being non-toxic to normal, i.e. primary and non-transformed cells. These findings strongly suggest a potential of this micronutrient in the therapy and/or prevention of cancer without significant side-effects. © 2001 Cancer Research Campaign http://www.bjcancer.com

**Keywords**: vitamin E analogue; malignant cells; apoptosis; selectivity

Cancer is one of the most common causes of mortality, hence the search for anticancer drugs has been intense. At present, most substances, whether used in clinical applications or in experimental settings, are relatively non-specific, often exerting adverse effects on normal cells. For instance, the established and widely used chemotherapeutic drug adriamycin (doxorubicin) is associated with cardiomyopathy consisting of congestive heart failure and dysrhythmias, probably due to toxic effects on cardiomyocytes (Henderson and Frei, 1980). Inducers of apoptosis, secreted by cells of the immune system, including the tumour necrosis factor-related apoptosis-inducing ligand, are toxic towards normal cells, such as hepatocytes (Jo et al, 2000).

We have observed recently that α-tocopheryl succinate (α-TOS), an esterified vitamin E analogue lacking the antioxidant activity of α-tocopherol, induced apoptosis in Jurkat T lymphoma cells (Neuzil et al, 1999). More recently, we found α-TOS to be pro-apoptotic for human colon cancer cells while normal skin fibroblasts were resistant (Neuzil et al, 2001). Together with data by others (Jha et al, 1999) suggesting a selective toxicity of α-TOS for malignant cells, these findings prompted us to systematically investigate this potentially important feature. Here we show that α-TOS preferentially kills malignant cells while showing very limited or no toxicity towards normal cells.

### MATERIALS AND METHODS

### Cell culture

Suspension cells were maintained in RPMI-1640 supplemented with 10% FCS plus antibiotics, adherent cells were grown in DMEM plus 10% FCS and antibiotics, while endothelial cells were cultures in endothelial cell medium with growth factors (Promo Cell) and antibiotics.

*Received 6 June 2000*
*Revised 8 September 2000*
*Accepted 18 September 2000*

*Correspondence to:* J Neuzil. E-mail: jneuzil@klp.med.uni-muenchen.de or C Weber. E-mail: cweber@klp.med.uni-muenchen.de

### Apoptosis induction and assessment

Apoptosis was induced by addition of 50 µM (final concentration) of α-TOS dissolved in DMSO, controls were treated with DMSO only. For suspension cells, cell density was $0.5 \times 10^6$ per ml at the onset of treatment, malignant adhesion cells were treated when 70–80% confluent, while normal adhesion cells were left to grow to confluency before treatment.

Apoptosis was assessed in suspension cells by the annexin V-FITC method as described earlier (Neuzil et al, 1999) and in adherent cells by the TUNEL-FITC method according to the manufacturer's protocol (Boehringer, Germany). The percentage of annexin V-positive cells was determined by flow cytometry (Becton Dickinson) and that of TUNEL-FITC-positive cells by scoring at least 100 cells in a fluorescent microscope.

### RESULTS AND DISCUSSION

We studied the effect α-TOS in a wide range of cell lines and normal cells. Strikingly, all cell lines tested were susceptible to α-TOS, as treatment with a pharmacological dose of 50 µM for 12 h triggered apoptosis in 28–65% of the cells (Table 1). In general, leukaemic cell lines, including monocytic and macrophage cell lines, B and T lymphoma cells, were more sensitive towards α-TOS than adherent carcinoma cell lines. Notwithstanding, α-TOS was pro-apoptotic towards lung, breast and colorectal cancer cells. Moreover, α-TOS efficiently killed p53- and $p21^{Waf1/Cip1}$-deficient mutants of the HCT-116 colon cancer cell line (not shown). Since mutations of tumour suppression genes are frequent complications in cancer chemo- and radiotherapy, this highlights the potential of α-TOS as an anticancer agent (Kaelin, 1999).

In contrast, normal cell types, including haematopoietic cells, fibroblasts, endothelial cells, cardiomyocytes, hepatocytes and smooth muscle cells, showed no susceptibility to apoptosis induced by α-TOS. This can be exemplified by cardiomyocytes where the effects of pro-apoptotic stimuli on morphology, cytoskeleton and beating rate were compared. While α-TOS had no effect, adriamycin induced morphological and cytoskeletal changes and

**Table 1** α-TOS induces apoptosis in transformed but not normal cells

| Cell type | Apoptosis[a] | |
|---|---|---|
| | Control | α-TOS |
| **Haematopoietic cell lines** | | |
| NSF/N1.H7 | 5.7 ± 3.2 | 36.4 ± 4.8 |
| HL-60 | 8.1 ± 2.6 | 52.2 ± 7.3 |
| THP-1 | 5.1 ± 2.8 | 48.8 ± 8.9 |
| U937 | 8.5 ± 3.8 | 59.6 ± 7.9 |
| J774 | 8.1 ± 3.3 | 65.4 ± 9.2 |
| Mono Mac 6 | 6.2 ± 2.1 | 54.7 ± 7.6 |
| Jurkat | 8.9 ± 3.1 | 45.2 ± 6.8 |
| K-562 | 4.5 ± 1.4 | 37.7 ± 5.2 |
| HPB | 6.5 ± 2.1 | 35.8 ± 4.7 |
| REH | 7.2 ± 3.8 | 46.9 ± 6.5 |
| CEM | 6.5 ± 2.1 | 44.8 ± 7.8 |
| Raji | 8.2 ± 2.1 | 56.3 ± 8.5 |
| Nalm-6 | 6.9 ± 3.5 | 49.6 ± 6.8 |
| **Adenocarcinoma lung cell line** | | |
| A549 | 1.1 ± 0.5 | 28.1 ± 4.2 |
| **Breast carcinoma cell line** | | |
| MCF-7 | 12.3 ±1.5 | 36.3 ± 7.3 |
| **Bronchocarcinoma cell line** | | |
| BEAS-2B | 1.9 ± 1.1 | 36.2 ± 5.9 |
| **Colon carcinoma cell lines** | | |
| CaCo-2 | 6.5 ± 2.2 | 63.1 ± 9.8 |
| HCT-116 | 7.5 ± 1.1 | 37.3 ± 4.8 |
| HCT-15 | 3.5 ± 1.6 | 32.3 ± 4.5 |
| DKO-1 | 6.5 ± 3.1 | 45.2 ± 6.3 |
| DKO-3 | 6.1 ± 4.5 | 43.2 ± 7.1 |
| DKS-5 | 5.5 ± 3.1 | 41.2 ± 6.4 |
| DKS-8 | 7.2 ± 1.9 | 37.2 ± 5.8 |
| DLD-1 | 5.2 ± 1.8 | 32.3 ± 5.3 |
| LS1034 | 4.2 ± 2.6 | 35.9 ± 6.9 |
| **Normal cells** | | |
| Mouse peritoneal macrophages | 2.1 ± 1.1 | 5.5 ± 3.4 |
| Human peripheral monocytic cells | 3.5 ± 2.8 | 6.2 ± 4.1 |
| Human monocyte-derived macrophages | 2.9 ± 1.5 | 5.2 ± 1.5 |
| Human skin fibroblasts[b] | 3.5 ± 0.9 | 6.1 ± 2.5 |
| Human foreskin fibroblasts | 3.8 ± 3.2 | 6.5 ± 4.7 |
| Human umbilical vein endothelial cells | 3.1 ± 1.2 | 6.5 ± 4.2 |
| Rat intestinal epithelial cells | 1.8 ± 1.6 | 3.5 ± 4.8 |
| Rat neonatal cardiomyocytes | 1.1 ± 0.9 | 4.2 ± 3.1 |
| Rat neonatal hepatocytes | 2.5 ± 1.9 | 4.3 ± 3.2 |
| Rat smooth muscle cells | 2.5 ± 1.8 | 4.9 ± 3.2 |

[a]Apoptosis was assessed following 12-h exposure of the cells to 50 µM α-TOS. For adherent cells, the extent of apoptosis is expressed as a sum of the corresponding values for cells that remained attached and those that detached during the experiment. Apoptosis extent was assessed by the annexin V method for suspension cells and TUNEL staining for adherent cells, and is expressed in % apoptotic cells as mean ± SD ($n$ = 3–6). [b]Normal proliferating adherent cells were left to reach confluence before treatment decreased beating rate (not shown). These findings are complemented by reports which showed no toxic effect of α-TOS on normal fibroblasts (Jha et al, 1999) or prostate cells (Israel et al, 2000), and by a finding that α-TOS was toxic towards malignant but protective for normal stem cells (Fariss et al, 1994).

The reasons for the selectivity of α-TOS are not known at present. However, the fact that it appears to affect rapidly proliferating cells is suggestive of a role of the cell cycle in sensitivity of malignant cells to the vitamin E analogue. We have found that pro-apoptotic activity of α-TOS positively correlated with its inhibitory activity on protein kinase C (PKC) (Neuzil et al, 2001), and PKC activity has been associated with a more proliferative and invasive phenotype of malignant cells, as shown e.g. for breast (Ways et al, 1995) or renal carcinoma cells (Engers et al, 2000). Moreover, PKC is also involved in fast transition through the cell cycle by regulating the cell cycle checkpoint proteins $p21^{Waf1/Cip1}$ and $p27^{Kipl}$ (Frey et al, 1997), and α-TOS has been shown to decrease levels of the cdk2-cyclin A complex and increased $p21^{Waf1/Cip1}$ in a human breast cancer cell line (Turley et al, 1997). Similarly, treatment of colon cancer cells with Trolox, a water-soluble analogue of vitamin E, led to the expression of $p21^{Waf1/Cip1}$ and sensitization of the cells towards 5-fluorouracil (Chinery et al, 1997). Notably, we have found that normal adherent cells which were confluent and thus contact-arrested and quiescent were resistant to α-TOS-induced toxicity (not shown).

 *© 2001 Cancer Research Campaign*

In conclusion, our results strongly suggest that α-TOS, a pharmacologically relevant micronutrient without known side-effects (Bendich and Machlin, 1988), is a potent pro-apoptotic agent for a variety of malignant cells, while being non-toxic for normal cells, and warrants testing as an anticancer drug or adjuvant in experimental animal models of tumorigenesis and leukaemia. This notion is further encouraged by recent findings that α-TOS inhibited tumour growth in nude mice with colon-cancer xenografts, similarly or more potently than did other experimental or established anticancer agents (Neuzil et al, 2001; Chinery et al, 1997). Furthermore, free vitamin E or α-tocopheryl acetate, the usual pharmacological form of vitamin E, do not have pro-apoptotic activity (Quian et al, 1997; Neuzil et al, 1999). Hence, our results may have important implications for therapy and prevention of cancer.

### REFERENCES

Bendich A and Machlin LJ (1988) Safety of oral intake of vitamin E. *Am J Clin Nutr* **48**: 612–619

Chinery R, Brockman JA, Peeler Mo, Shyr Y, Beauchamp RD and Coffey RJ (1997) Antioxidants enhance the toxicity of chemotherapeutic agents in colorectal cancer: A p53-independent induction of p21$^{Waf1/Cip1}$ via C/EBP β. *Nature Med* **3**: 1233–1241

Engers R, Mrzyk S, Springer E, Fabbro D, Weissgerber G, Gernharz CD and Gabbert HE (2000) Protein kinase C in human renal cell carcinomas: role in invasion and differential isoenzyme expression. *Br J Cancer* **82**: 1063–1069

Fariss MW, Fortuna MB, Everett CK, Smith JD, Trent DF and Djuric Z (1994) The selective antiproliferative effects of α-tocopheryl hemisuccinate and cholesteryl hemisuccinate on murine leukemia cells result from the action of the intact compounds. *Cancer Res* **54**: 3346–3351

Frey MR, Saxon ML, Zho X, Rollins A, Evans SS and Black JD (1997) Protein kinase C isozyme-mediated cell cycle arrest involves induction of p21$^{Waf1/Cip1}$ and p27$^{Kip1}$ and hypophosphorylation of the retinoblastoma protein in intestinal epithelial cells. *J Biol Chem* **272**: 9424–9435

Henderson IC and Frei E (1980) Adriamycin cardiotoxicity. *Am Heart J* **99**: 671–674

Israel K, Yu W, Sanders BG and Kline K (2000) Vitamin E succinate induces apoptosis in human prostate cancer cells: role for Fas in vitamin E succinate-triggered apoptosis. *Nutr Cancer* **36**: 90–100

Jha MN, Bedford JS, Cole WC, Edward-Prasad J and Prasad KN (1999) Vitamin E (d-α-tocopheryl succinate) decreases mitotic accumulation in gamma-irradiated human tumor, but not in normal cells. *Nutr Cancer* **35**: 189–194

Jo M, Kim TH, Seol DW, Esplen JE, Dorko K, Billiar TR and Strom SC (2000) Apoptosis induced in normal human hepatocytes by tumor necrosis factor-related apoptosis-inducing ligand. *Nature Med* **6**: 564–567

Kaelin WG (1999) Choosing anticancer drug targets in the postgenomic era. *J Clin Invest* **104**: 1645–1506

Neuzil J, Svensson I, Weber T, Weber C and Brunk U (1999) α-Tocopheryl succinate-induced apoptosis in Jurkat T cells involves caspase-3 activation, and both lysosomal and mitochondrial destabilisation. *FEBS Lett* **445**: 295–300

Neuzil J, Weber T, Schroeder A, Lu M, Ostermann G, Gellert N, Mayne GC, Olejnicka B, Negre-Salvayre A, Sticha M, Coffey RJ and Weber C (2001) Induction of cancer cell apoptosis by α-tocopheryl succinate: molecular pathways and structural requirements. *FASEB J*: in press

Qian M, Kralova J, Yu W, Bose HR, Dvorak M, Sanders BG and Kline K (1997) c-Jun involvement in vitamin E succinate induced apoptosis of reticuloendotheliosis virus transformed avian lymphoid cells. *Oncogene* **15**: 223–230

Turley JM, Ruscetti FW, Kim SJ, Fu T, Gou FV and Birchenall-Roberts MC (1997) Vitamin E succinate inhibits proliferation of BT-20 human breast cancer cells: increased binding of cyclin A negatively regulates E2F transactivation activity. *Cancer Res* **57**: 2668–2675

Ways DK, Kukoly CA, deVente J, Hooker JL, Bryant WO, Posekany KJ, Fletscher DJ, Cook PP and Parker PJ (1995) MCF-7 breast cancer cells transfected with protein kinase C-α exhibit altered expression of other protein kinase C isoforms and display a more aggressive neoplastic phenotype. *J Clin Invest* **95**: 1906–1915

© 2001 Cancer Research Campaign

